# A reanalysis dataset of the South China Sea

**DOI:** 10.1038/sdata.2014.52

**Published:** 2014-12-23

**Authors:** Xuezhi Zeng, Shiqiu Peng, Zhijin Li, Yiquan Qi, Rongyu Chen

**Affiliations:** 1 State Key Laboratory of Tropical Oceanography, South China Sea Institute of Oceanology, Chinese Academy of Sciences, Guangzhou 510301, China; 2 University of Chinese Academy of Sciences, Beijing 100049, China; 3 Jet Propulsion Laboratory, California Institute of Technology, Pasadena, California 91109, USA

## Abstract

Ocean reanalysis provides a temporally continuous and spatially gridded four-dimensional estimate of the ocean state for a better understanding of the ocean dynamics and its spatial/temporal variability. Here we present a 19-year (1992–2010) high-resolution ocean reanalysis dataset of the upper ocean in the South China Sea (SCS) produced from an ocean data assimilation system. A wide variety of observations, including *in-situ* temperature/salinity profiles, ship-measured and satellite-derived sea surface temperatures, and sea surface height anomalies from satellite altimetry, are assimilated into the outputs of an ocean general circulation model using a multi-scale incremental three-dimensional variational data assimilation scheme, yielding a daily high-resolution reanalysis dataset of the SCS. Comparisons between the reanalysis and independent observations support the reliability of the dataset. The presented dataset provides the research community of the SCS an important data source for studying the thermodynamic processes of the ocean circulation and meso-scale features in the SCS, including their spatial and temporal variability.

## Background & Summary

A long-term high-quality dataset with high spatial resolution and temporal continuity is required for studying the thermodynamic processes of the ocean state as well as their spatial and temporal variability. Although sampling from ships and buoys in the ocean continue to increase, such *in-situ* observations are still spatially sparse and temporally discontinuous. Advances in satellite technology during the past several decades have facilitated ocean observing with better spatial and temporal coverage, but satellites only provide the surface observations of the ocean such as sea surface height anomalies (SSHAs), sea surface temperatures (SSTs) and sea surface salinities (SSSs). The gridded model outputs, on the other hand, are of higher spatial resolution and temporal continuity; however, they may deviate from the ‘true’ ocean state considerably and contain large uncertainties. Ocean reanalysis is thus a ‘necessary’ choice that combines historical ocean observations with a general circulation oceanic model via a data assimilation algorithm to reconstruct a long time series of historical three-dimensional ocean states. It overcomes the shortcomings of sparse spatial and temporal distributions in observations and provides a better estimate of the ocean than obtained only by modeling or by sparse observations. Ocean reanalysis can serve for studying complex spatial structures such as from meso-scale to large-scale and temporal evolutions from days to years of the oceanic variables, as well as the physical relationship between different variables and dynamic processes of oceanic phenomena^1–3^. It can also be used for the lateral boundary or initial conditions for model simulation and prediction^4^.

Many ocean reanalysis projects have been carried out worldwide, such as SODA^5,6^ (Simple Ocean Data Assimilation), ECCO^7^ (Estimating the Circulation and Climate of the Ocean), NCEP-GODAS^8^ (Global Ocean Data Assimilation System), HYCOM^9^ (Hybrid Coordinate Ocean Model) and CORA (China Ocean Reanalysis). Particularly, the CORA, consisting of a global version^10^ and a regional version^11,12^, is focused on coastal waters of China and its adjacent seas.

**Figure 1:**
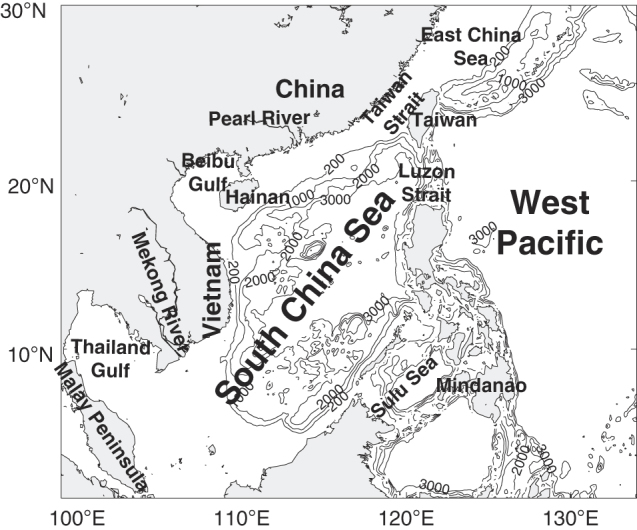
Model domain and bathymetry (unit: m).

As the largest marginal sea in the tropics with a maximum depth of more than 5,000 m, the South China Sea (SCS) plays a vital role in the water mass and energy exchange between the Pacific and Indian Oceans^13^. As a part of the Indo-Pacific warm pool, the SCS also has a significant influence on the regional climate change^14^. It features a broad varying bathymetry from tens of meters at continental shelves to more than 5,000 m at the center (Figure 1), and complex seasonal-varying circulations associated with East Asia monsoons, including intensified western boundary currents, throughflows, gyres and meso-scale eddies. Therefore, the SCS has been given increasing attention by researchers recently. However, to our knowledge, there is no a high-resolution reanalysis dataset which mainly focuses on the SCS yet. Although the global reanalysis datasets or some of the regional reanalysis datasets mentioned above cover the SCS region, they generally provide an ocean state estimate on a grid of 1/2° to 1/3°, which is inadequate for studying the ocean meso-scale processes. The global reanalysis HYCOM has a spatial resolution of 1/12°; however, it may contain large uncertainties in the SCS because it does not focus on this region and thus assimilates less observations of the region.

In the past several decades, mainly sponsored by the South China Sea Institute of Oceanology (SCSIO) through a number of various projects such as the Northern South China Sea Open Cruise (NSCSOC) supported by the National Science Foundation of China (NSFC), seasonally regular and feature-targeted surveys have been conducted to observe the marine environment over the SCS. A significant number of temperature/salinity (T/S) profiles have been collected annually. These multi-year accumulated T/S profiles, together with satellite-derived SSTs and SSHAs, make it possible to construct a high-resolution ocean reanalysis of the SCS. Using a multi-scale incremental three-dimensional variational data assimilation method^15,16^, these observations are assimilated into the outputs of a high-resolution model called the Regional Oceanic Modeling System^17,18^ (ROMS), yielding a reanalysis dataset of the SCS (called REDOS for short hereafter). Here we present the gridded dataset of daily estimates with a horizontal resolution of 0.1°×0.1° from the sea surface to 1,200 m water depth in the SCS and its adjacent area, which contains most of the physical-oceanography variables including the temperature (T), salinity (S), zonal (U) and meridional (V) components of current velocity, and sea surface height (SSH). The purpose of REDOS is to provide 1) a better estimate than obtained only by sparse observations or models and 2) a temporally continuous and spatially gridded data source with high resolution and high quality for research community to study the thermodynamic processes of the SCS as well as their spatial/temporal variability.

## Methods

The reanalysis system used for producing REDOS consists of three components: the regional ocean model, data assimilation algorithm and quality control for observations.

### Model and configuration

ROMS is employed as the regional ocean model in this ocean reanalysis system. The model domain covers the whole SCS and its adjacent area from 1°N to 30°N, 99°E to 134°E, with an eddy-resolved horizontal resolution of 0.1°×0.1° (about 10 km) and 32 S-coordinate layers in the vertical (Figure 1). The parameterization of the vertical mixing process adopts the K-profile parameterization (KPP) scheme^19^. The bathymetry used in the model is produced using the ETOPO1^20^ data. The minimum and maximum depths in the whole domain are set to be 10 and 5,000 m, respectively. The wind field employed is a daily mean wind field from the Cross-Calibrated Multi-Platform (CCMP) ocean surface wind product^21^ with a horizontal resolution of 0.25°×0.25°, which is converted into the wind stress using the bulk formula given by *Large and Pond*^22^. The other daily atmospheric forcing fields, including heat fluxes, solar radiation fluxes, Evaporation-Precipitation (E-P), air temperature and specific humidity, are obtained from the U.S. National Centers of Environmental Prediction (NCEP) reanalysis^23^ with a horizontal resolution of 1.875°×1.875°. Monthly temperature, salinity, sea surface height and velocity field from SODA provide the lateral boundary conditions. The World Ocean Altas 01 (WOA01)^24^ monthly climatology of temperature and salinity is used to initialize for spin-up and a spin-up of 10 years is performed. River runoff and tides are not considered in the reanalysis system currently. The datasets used for forcing the model at initial time and upper/lateral boundaries are summarized in Table 1 (available online only).

### Data assimilation algorithm

**Figure 2:**
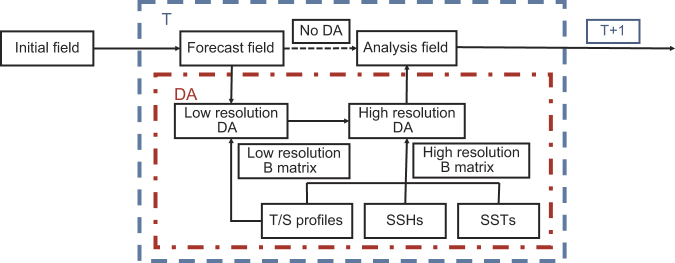
The flow schematic of the data assimilation system.

A multi-scale incremental three-dimensional variational data assimilation (3DVAR) scheme developed by Li *et al.*^15,16^ is used to assimilate the observations into ROMS. Figure 2 shows a flow chart of the data assimilation strategy employed in the reanalysis system. The features of the data assimilation scheme are summarized as follows:

(1) It is able to assimilate multi measurements, such as SSTs, SSHs, sea surface velocities (SSVs) and T/S profiles separately or simultaneously.

(2) It assimilates the observations at two spatial scales for taking into account the large- and small-scale information represented by different sampling density of different observation types, e.g., the sparse T/S profiles and ship-track SSTs and the dense satellite-derived SSHAs and SSTs. Specifically, the cost function it minimizes is divided into two parts as follows:

(1)JL(δxL)=12δxLTBL−1δxL+12(HδxL−δy)T(R+HBSHT)−1(HδxL−δy)

(2)JS(δxS)=12δxSTBS−1δxS+12(HδxS−δy)T(R+HBLHT)−1(HδxS−δy)

Where the subscripts *L* and *S* denote large- and small-scale, respectively; and the superscript *T* denotes transpose operator. *δ***x**=**x−x**^b^ is the increment measuring the deviation of optimal values of model variable vector **x** from their corresponding forecasted values **x**^b^; *δ**y***=***y*****−*****H*****x**^b^ is the innovation measuring the deviation of observations *y* from their corresponding model output *H***x**^b^; **H** is the Jacobian matrix of the nonlinearly observational operator; **B** and **R** are the background error covariance matrix and observational error covariance matrix, respectively. It is worth noting that the small-scale background error covariance matrix **B**_*S*_ is included in the observation errors in the form of representativeness error (**HB**_*S*_**H**^*T*^) in the large-scale cost function (1), and *vice versa*. Such a deliberately designed scheme makes it possible to reduce or eliminate the representativeness error, thus improve the effectiveness of the high-resolution data assimilation.

(3) The control variables, including SSH, streamfunction, velocity potential, temperature and salinity, are expressed in the form of increments. SSH increments are decomposed into the steric and non-steric parts, associated with the balance between temperature and salinity and the water mass convergence of a water column, respectively. Likewise, the current increments are decomposed into the geostrophic and ageostrophic components, and the incremental ageostrophic velocity is expressed in the form of the incremental ageostrophic streamfunction and velocity potential.

(4) For computational efficiency, data assimilation is carried out on 24 standard z layers from the sea surface to 1,200 m water depth, instead of on vertical S-coordinate layers.

The most important element in a data assimilation system is the background error covariance matrix (**B**), which determines the correlation relationship between different variables and the spatial influencing scale of the observations. The construction of **B** requires the knowledge of the background errors and the horizontal/vertical correlations between different control variables for all grids. Here we use the so-called NMC method^25^ to generate the proxy of background errors from a 19-year simulation. A Gaussian correlation model **C**^*x/y*^(*r*)=exp[−*r*^2^/(2*L*^2^)] is used to compute the horizontal correlation by assuming that it is homogeneous and isotropic^26^; the horizontal correlation length scale *L*, estimated from the proxy of background errors, is set to be 70 km and 300 km for the high- and low- resolution grids, respectively.

Due to the existence of the thermocline and halocline, it is inappropriate to use an analytical function to compute the vertical correlation matrix **C**^*z*^. Fortunately, the size of **C**^*z*^ (*n*_*z*_×*n*_*z*_ with *n*_*z*_=24 in this system) is much smaller compared to that of the horizontal correlation matrix **C**^*x/y*^ and thus is tractable. Therefore, we compute **C**^*z*^ directly from the proxy of background errors. In addition, considering the different characteristics of the water masses in the SCS and West Pacific (WP), **C**^*z*^ is computed separately for these two regions. The vertical standard deviations of temperature and salinity needed for constructing **B** are also directly computed from the proxy of background errors for the SCS and WP, respectively.

### Observations and quality control

The observations assimilated into the reanalysis system include SSTs, SSHAs and *in-situ* T/S profiles (see Table 1 (available online only)). SST data is from Advanced Very High Resolution Radiometer (AVHRR) Pathfinder Version 5.2^27^ from 1992 to 1998 and Multi-Channel Sea Surface Temperature (MCSST, http://www.usgodae.org) from 1999 to 2010. MCSST is also AVHRR SST retrievals but under the quality control (QC) by the Fleet Numerical Meteorology and Oceanography Center (FNMOC). SSHA data is the delayed-time along-track product by AVISO. Details description can be found in SSALTO/DUACS User Handbook^28^. *In-situ* T/S profiles are from a variety of sources, including Argo (Array for Real-time Geostrophic Oceanography, http://www.argo.ucsd.edu), WOD09^29^ (World Ocean Database 2009), CTD and XBT data from the cruises carried out by SCSIO.

**Figure 3:**
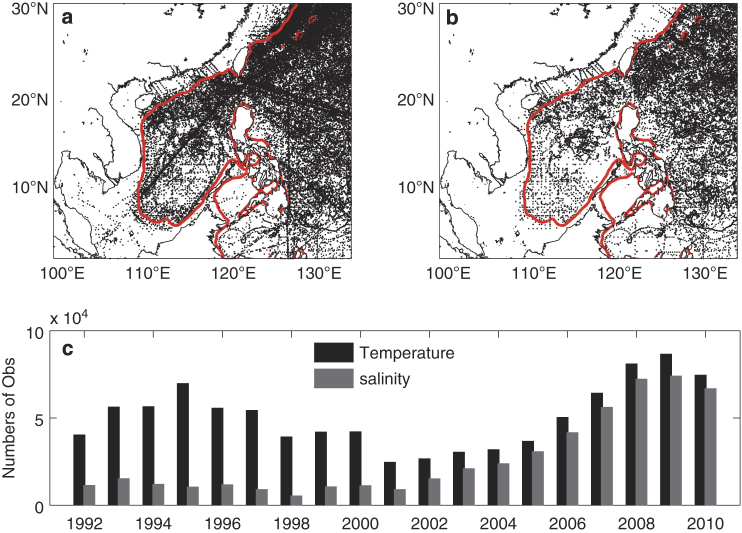
Statistics of the temperature and salinity profiles. Spatial distributions of (**a**) temperature and (**b**) salinity profiles and (**c**) the temporal evolutions of temperature and salinity observations from 1992 to 2010 within the model domain. The red contours in (**a**) and (**b**) are the 200 m isobaths. The profiles shown in (**a**) and (**b**) are those passing QC, while the observation numbers shown in (**c**) are those passing QC and interpolated into the model vertical levels.

QC is an important part of a data assimilation system. Observations with poor quality may offset the positive effect of good observations on the reanalysis or cause non-convergence of data assimilation procedure, thus should be excluded before performing data assimilation. SSTs and SSHAs from satellites and T/S profiles from Argo are quality controlled by the corresponding organizers who released them. Although particular care was brought to process SSHA data near coasts^30^ where the satellite swath could be contaminated severely, the data in shallow waters (depth less than 200 m) are not used. Following *Ingleby and Huddleston*^31^, several checks, including checks for sea/land, range, stability, and spike, are performed to ensure the quality of T/S profiles from WOD09 and cruises, which could contain considerable errors due to their multi-source and manual operations. Then a duplication check is performed to remove the duplicate data in all the T/S profiles. After such a procedure of QC, the number of T/S profiles within the model domain is 55,398, accounting about 72% of the total T/S profiles. Ninety percent of the T/S profiles passing QC are used for data assimilation which are then interpolated onto the standard z layers for input to the reanalysis configuration (Figure 3), and the rest are retained for verification.

### Generation of the reanalysis data

REDOS is produced following a two-step procedure. Firstly, a 10-year spin-up run of ROMS, initialized with WOA01 climatological monthly temperature and salinity and forced by climatological monthly atmospheric fields (as listed in Table 1 (available online only)), is carried out to obtain an ocean state of dynamic balances among the variables. Secondly, initialized with the balanced ocean state from the spin-up run and forced by the daily atmospheric fields, the model runs from 1992 to 2010 and observations are assimilated into the model once a day using the multi-scale incremental 3DVAR algorithm, finally yielding the 19-year high-resolution REDOS.

### Code availability

Source code of the data assimilation system will be provided upon request for the purpose of replicating the reanalysis data described in this paper. Code may be requesting by email from the corresponding author.

## Data Records

REDOS contains 19x365 daily data files (one data file per day) in the form of NetCDF that is available from the figshare record associated with this publication (Data Citation 1). The fields contained in each data file include:

1. lon: longitudes for T, S and SSH;
2. lat: latitudes for T, S and SSH;
3. lon_u: longitudes for zonal component of current;
4. lat_u: latitudes for zonal component of current;
5. lon_v: longitudes for meridional component of current;
6. lat_v: latitudes for meridional component of current;
7. depth: vertical layers of all kinds of variables;
8. t: temperature;
9. s: salinity;
10. u: zonal component of current;
11. v: meridional component of current;
12. zeta: sea surface height.

## Technical Validation

REDOS is mainly evaluated by comparing with independent observations and other ocean reanalysis data.

### Temperatures and salinities

**Figure 4:**
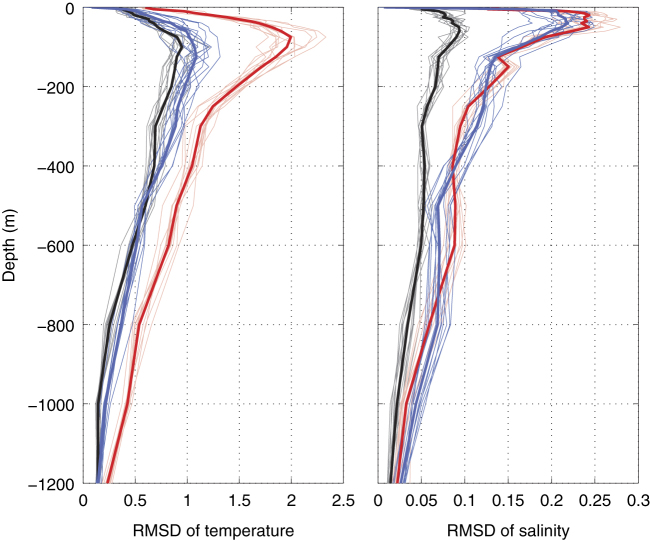
Validation of the temperature and salinity of REDOS (HYCOM) against the independent observations. Vertical profiles of root mean square differences (RMSDs) for temperature (left panels, unit: °C) and salinity (right panels, unit: psu) for REDOS (black lines), free model simulation (red lines) and HYCOM (blue lines) for each (thin lines) and the mean (thick lines) of 12 months.

To evaluate the estimates of temperature and salinity in REDOS, we calculated the root mean square differences (RMSDs) of temperature and salinity against over 2,000 independent samples of T/S profiles, which are excluded from the data assimilation procedure of REDOS (Figure 4). The averaged RMSD at each layer is less than 1 °C (0.1 psu) for temperature (salinity) estimates in REDOS, with a maximum of ~1.2 °C (~0.12 psu) located at the seasonal thermocline (halocline) and a vertical mean of 0.60 °C (0.06 psu). The seasonal thermocline and halocline are also the area difficult to simulate in an ocean general model. In contrast, the vertically averaged and maximum RMSDs of temperature (salinity) from free model run (i.e., without data assimilation) reaches up to 1.24 °C (0.16 psu) and 2 °C (0.24 psu), respectively, as twice as those in REDOS. Compared with the high-resolution analysis dataset HYCOM with a vertical mean of ~0.69 °C (0.14 psu) and maximum RMSDs of ~1.1 °C (0.22 psu), REDOS obviously improves the estimates of the temperature and salinity up to 1,200 m water depth, especially for the salinity which shows large bias in HYCOM even comparable to that of free model run of ROMS. This is probably due to the much more *in-situ* observations being assimilated in REDOS than those in HYCOM as well as the employed multi-scale data assimilation scheme with a specific background error covariance solely designed for the SCS region.

**Figure 5:**
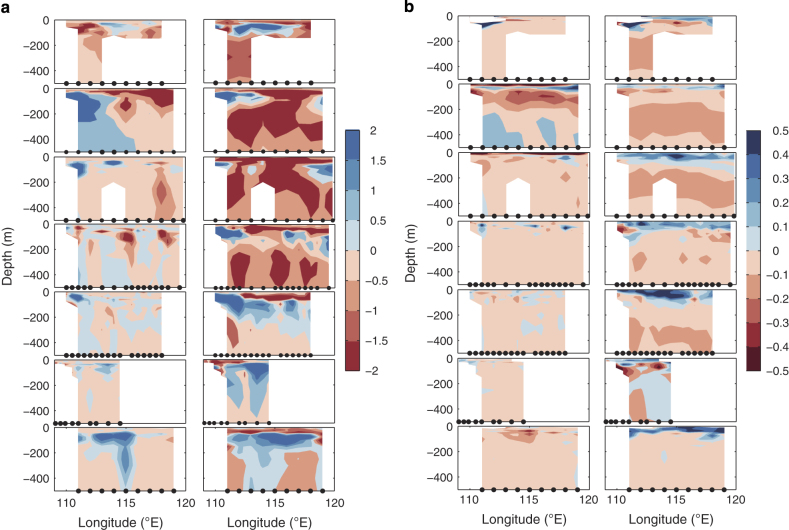
Validation of temperature and salinity of REDOS (HYCOM) against *in-situ* observations. Vertical distributions of the differences of temperature (**a**, unit: °C) and salinity (**b**, unit: psu) between REDOS and observations (left panels in (**a**) and (**b**)) and the ones between HYCOM and observations (right panels in (**a**) and (**b**)) from 2004 to 2010 (from upper to bottom). The black dots denote the observation sites on the 18°N transect of the Northern South China Sea Open Cruise (NSCSOC).

The NSCSOC field campaign carried out by the SCSIO has taken a regular survey on the 18°N transect since 2004 and obtained multi-year observations of T/S profiles on the same transect. These observations are very valuable for both analyzing the evolution of the ocean state in the region and validating the accuracy of outputs from numerical models or data assimilation schemes. Here we validate REDOS against the multi-year (2004–2010) observations of the 18°N transect. Note that these observations are not independent validation samples as some of them have been assimilated into REDOS. As shown in Figure 5, except for 2005 which shows large biases both for temperature and salinity, the biases of temperature (salinity) of REDOS against observations are within the range of ±0.5 °C (±0.1 psu) in the upper 500 m on the 18°N transect. The largest temperature (salinity) biases are located at the seasonal thermocline (halocline) which is in agreement with the RMSD profiles shown in Figure 4. In addition, large salinity biases are also found near the sea surface in 2005 and 2006. Again, a comparison to the HYCOM dataset (right columns of Figure 5a,b) indicates that the biases of HYCOM are generally larger than those of REDOS, and the large biases are found not only at the seasonal thermocline/halocline but also beneath the mixing layer.

### Sea surface heights

Because of the different reference levels, SSHs from models are difficult to compare directly with observations, such as those from satellite altimetry and tide gauge records. Therefore, we mainly evaluate SSHs by examining the correlation between the reanalysis and observations.

**Figure 6:**
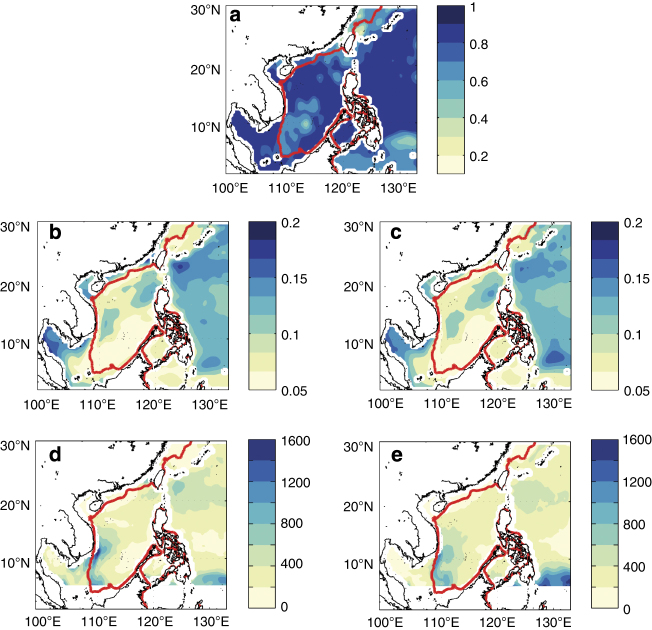
Validation of SSH of REDOS against altimetry data. (**a**) Correlation coefficients of sea surface heights (SSHs) between REDOS and altimetry data from October 1992 to December 2010; (**b** and **c**) Standard deviations (unit: m) of SSHs from altimetry data and REDOS, respectively; (**d** and **e**) Mean eddy kinetic energy (EKE, unit: cm^2^ s^−2^) from altimetry data and REDOS, respectively. The data within the 50 km offshore are neglected due to the bad quality of the altimetry data there. The data in the south of 5°N, where the flow do not meet the geostrophic approximation, are also neglected in (**d** and **e**). The red contours are the 100 m isobaths.

We first validate the reanalysis against the AVISO delayed-time daily gridded data. Figure 6a gives the simultaneous correlation coefficients between the REDOS SSHs and altimetric SSHs (altimetric SSHA plus a climatologic mean from a 19-year model run) from October 1992 to December 2010. In most regions, REDOS SSHs agree well with altimetric data (with correlation coefficients higher than 0.8, above 95% confidence level). Correlation reduces in eddy-rich regions, such as the regions of the Kuroshio, Mindanao, south of Vietnam and west of Luzon Strait, probably due to the relative high variability of SSH in these regions that are difficult to catch in the REDOS. The patterns of the standard deviations of the REDOS SSHs and altimetric SSHs agree quite well with each other, with high variations in the regions of the Thailand Gulf, Mindanao and Kuroshio (Figure 6b,c). A comparison of the mean eddy kinetic energy (EKE), which is deduced from sea surface height anomaly using the geostrophic approximation, is also given in Figure 6d,e. A 10-day low-pass filter is employed to remove the REDOS's high frequency oscillation, which is not represented in altimetry due to its longer sample interval. The results indicate the mean EKE from the REDOS and altimetry data share the similar pattern, with relative high value east off Vietnam, in the east of Luzon Strait and in the Mindanao area.

**Figure 7:**
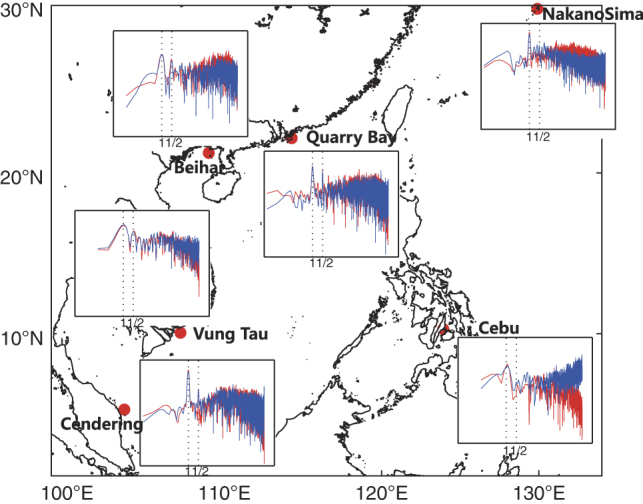
Validation of SSH of REDOS against tide gauge observations in power spectral density. Power spectral densities (PSDs) of sea surface heights for REDOS (blue lines) and tide gauge observations (red lines) from a few stations located in the SCS. Annual and semi-annual periods have been marked and the y-axis is in logarithmic form in each PSD figure. Linear trends have been removed from all records.

We next validate the reanalysis against some tide gauge records. University of Hawaii Sea Level Center (UHSLC, http://uhslc.soest.hawaii.edu) provides hundreds of tide gauge records all over the world. Thirty-three tide gauge records, identified by location (within our domain) and record length (exceeding two years in length), are chosen for validation. They are daily data with the diurnal cycle of the tides removed. Because the model in the reanalysis system does not include tides, the tide-filter as discussed by Xie *et al.*^32^ is not necessary to apply here and the comparison is straightforward. A linear trend is also removed to eliminate non-model effects such as sea level rise by ice melting in the tide gauge records and the model bias in the reanalysis. Correlation coefficients between observed and reanalysis sea levels are presented in Table 2 (available online only). Power spectral densities (PSDs) of SSHs are also given in Figure 7. The overall correlations (Cor1) are higher than 0.5 at all of the stations except Kaohsiung (0.42, south of Taiwan), Beihai (0.41, inside the Beibu Gulf) and Cebu (0.49, between Sulu Sea and WP). In particular, it is astonishing that correlations (Cor1) for Vung Tau (near Mekong River Estuary), Ko Lak (inside the Thailand Gulf) and the stations at east coast of Malay Peninsula (including Geting, Cendering, Kuantan, Tioman and Sedili) are above 0.9. The PSDs for all stations indicate that strong annual cycle exists in the tide gauge records and the reanalysis well reproduces this signal (as examples, Figure 7 shows the results of a few stations). In order to evaluate how well the reanalysis represents the observed sea levels at other frequency bands, we next remove this dominating annual cycle in both the reanalysis and tide gauge records. The correlation coefficients after isolating the annual cycle (Cor2 in Table 2 (available online only)) are significantly reduced for most of the stations, especially for the stations at the Kuroshio region (including Nase, Nakano Sima, Naha, Ishigaki, Keelung and Kaohsiung), where sea levels are dominated by the annual cycle (Figure 7). In particular, Cor2 for Cebu, a station at a narrow and shallow waterway connecting Sulu Sea and WP, is as low as 0.29. This low correlation is probably due to incapability of the REDOS in resolving the complex sea/land distribution and bathymetry there that leads to the exaggeration of the high frequency variations of SSHs in the REDOS. Except for this station, Cor2 for most of the stations in the SCS remains above 0.4. Besides the annual cycle, a semi-annual cycle of SSHs is also obvious in the observations (Figure 7), probably caused by the annual-reversed monsoons prevailing in the SCS. The reanalysis well reproduce this semi-annual cycle as well as higher frequency variations of SSHs for most of the stations in the SCS (Figure 7). In addition, it is worthy to note that correlation coefficients at two stations (Beihai and Kanmen) increase after filtered.

In general, the validations against the two observed datasets (satellite altimetry and tide gauge records) show a similar pattern of correlation, i.e., low correlation occurs around Taiwan Island and the Kuroshio region while high correlation is found in most region of the SCS.

### Currents

**Figure 8:**
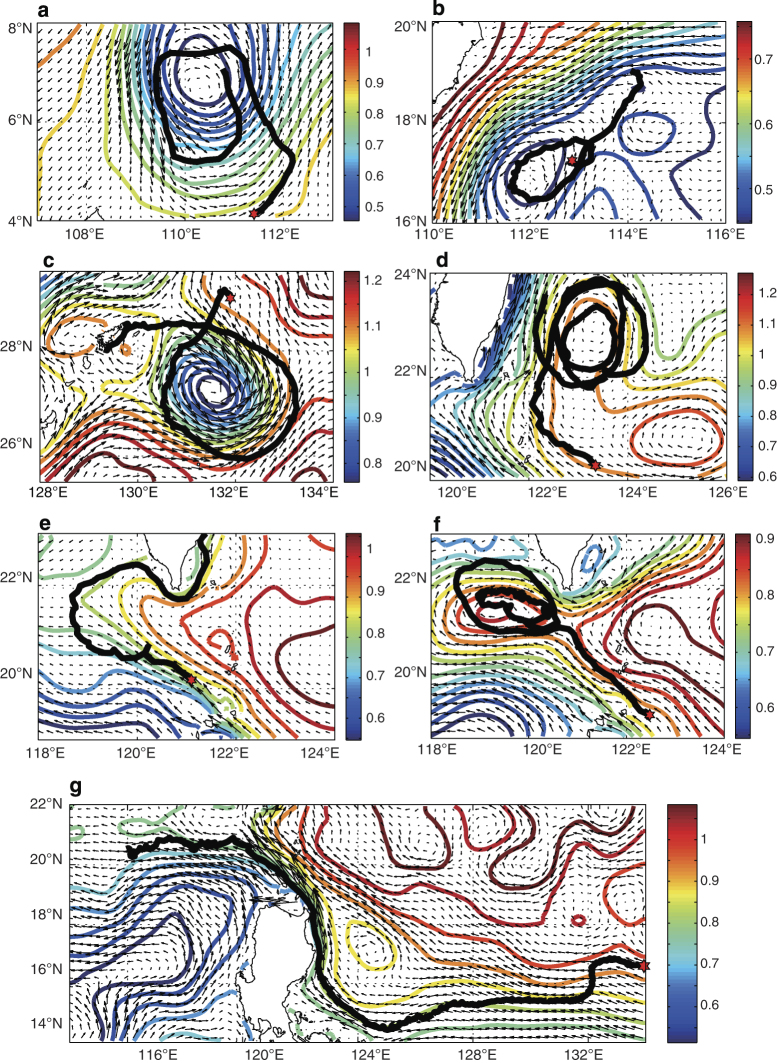
Validation of currents of REDOS against trajectories of drifting buoys. Seven drifting buoys are selected that were driven by the meso-scale eddies (**a**–**d**) or the Kuroshio intrusion into the SCS (**e**–**g**). Black thick lines depict the trajectories of drifting buoys starting from red hexagrams, while vectors (unit: m s^−1^) and contour lines (unit: m) denote current fields and sea surface heights of REDOS, respectively.

Because of large uncertainties in the modeled currents and rare observations for currents relative to other variables such as temperature, salinity and SSH, it is difficult to perform quantitative validation for currents, especially for currents in deep water. Therefore, first we give a qualitative validation to the near surface currents of REDOS by comparing them with the satellite-tracked drifting buoy observations provided by the Surface Velocity Program^33^. Several trajectories of drifting buoys, along with the current fields and sea surface heights from REDOS in the same period, are shown in Figure 8. It is found that, in general, the near surface currents estimated by REDOS well match the trajectories of drifting buoys in the strong current area. Particularly, the vortexes (eddies) depicted by drifting buoys in different parts of the SCS and WP, such as east of Vietnam (Figure 8a), east of Hainan Island (Figure 8b), Kuroshio region (Figure 8c) and east of Taiwan Island (Figure 8d), are well reproduced by REDOS. Noticeably, REDOS also well reproduces the different types of Kuroshio intrusion into the SCS, including a loop current intrusion (Figure 8e), an eddy shedding from a loop current (Figure 8f) and a direct intrusion across Luzon Strait (Figure 8g). A careful comparison of these trajectories to the current fields reveals that the trajectories and currents do not fit very well with each other in Figure 8a,f, which could be caused by the effects of downwind-slipping due to the loss of drogues in these drifters that are centered at the depth of 15 m beneath the sea surface.

In addition, a rough quantitative comparison between near-surface velocities from the REDOS and the drifter trajectories is given in Table 3 (available online only). Those drifter data that lost their drogues are discarded, because they are intensely affected by winds. As shown in Table 3 (available online only), the means of climatological monthly RMSDs of U-component, V-component and velocity magnitude are 19.51 cm s^−1^, 19.07 cm s^−1^ and 20.09 cm s^−1^, respectively.

## Additional information

Tables 1–3 are only available in online version of this paper.

**How to cite this article:** Zeng, X. *et al.* A reanalysis dataset of the South China Sea. *Sci. Data* 1:140052 doi: 10.1038/sdata.2014.52 (2014).

## Supplementary Material



## Figures and Tables

**Table 1:** Input datasets of the reanalysis system

**Data type**	**Variables**	**Source**	**Spatio-temporal resolution**
Initial condition	T, S	WOA01	0.25°×0.25°, climatological monthly
Boundary condition	T, S, U, V, and SSH	SODA	0.5°×0.5°, monthly
Topography	Gridded water depth	ETOPO1	1′×1′
Forcing	10-m Wind	CCMP	0.25°×0.25°, daily
	Heat flux	NCEP	1.875°×1.875°, daily
	Freshwater flux	NCEP	1.875°×1.875°, daily
Assimilated observations	T/S profile	Argo, WOD09, CTD and XBT data collected by SCSIO	Scatter data
	SST	AVHRR Pathfinder Version 5.2, MCSST, ship-track SST	Scatter data
	SSHA	AVISO	Along-track
*WOA01: World Ocean Altas 2001; SODA: Simple Ocean Data Assimilation; ETOPO1: 1 Arc-Minute Global Relief Model; CCMP: Cross-Calibrated Multi-Platform ocean surface wind product; NCEP: US National Centers of Environmental Prediction; Argo: Array for Real-time Geostrophic Oceanography, http://www.argo.ucsd.edu; WOD09: World Ocean Database 2009; AVHRR: Advanced Very High Resolution Radiometer; MCSST: Multi-Channel Sea Surface Temperature, http://www.usgodae.org/; CTD: Conductivity-Temperature-Depth; XBT: expendable bathythermograph.			

**Table 2:** Validation of sea surface height estimates in REDOS against several stations in the SCS

**Station Name**	**Location**	**Years**	**Cor1**	**Cor2**
South China Sea and East China Sea				
Shanwei	22.750°N, 115.350°E	6	0.65	0.54
Quarry Bay	22.600°N, 114.220°E	19	0.73	0.69
Zhapo	21.583°N, 111.833°E	6	0.67	0.60
Haikou	20.017°N, 110.283°E	6	0.60	0.54
Dongfang	19.100°N, 108.617°E	6	0.67	0.52
Beihai	21.483°N, 109.083°E	6	0.41	0.49
Qui Nhon	13.775°N, 109.254°E	3	0.78	0.72
Vung Tau	10.330°N, 107.070°E	3	0.92	0.82
Ko Lak	11.800°N, 99.820°E	18	0.91	0.75
Geting	6.230°N, 102.110°E	15	0.90	0.44
Cendering	5.267°N, 103.183°E	15	0.94	0.46
Kuantan	3.983°N, 103.433°E	15	0.94	0.44
Tioman	2.800°N, 104.133°E	15	0.92	0.45
Sedili	1.933°N, 104.117°E	15	0.92	0.46
Keppel Harbour	1.270°N, 103.820°E	18	0.88	0.69
Bintulu	3.260°N, 113.060°E	13	0.76	0.56
Miri	4.400°N, 113.967°E	6	0.67	0.54
Kota Kinabalu	5.983°N, 116.067°E	13	0.75	0.63
Manila	14.640°N, 121.080°E	5	0.73	0.38
Subic Bay	14.817°N, 120.283°E	4	0.79	0.58
Kaohsiung	22.617°N, 120.283°E	19	0.42	0.25
Keelung	25.150°N, 121.750°E	14	0.53	0.21
Xiamen	24.450°N, 118.067°E	6	0.73	0.62
Kanmen	28.083°N, 121.280°E	6	0.51	0.58
West Pacific and Sulu Sea				
Nakano Sima	29.830°N, 129.850°E	19	0.55	0.13
Nase	28.383°N, 129.500°E	19	0.63	0.30
Naha	26.220°N, 127.670°E	19	0.61	0.36
Ishigaki	24.333°N, 124.170°E	19	0.54	0.29
Cebu	10.300°N, 123.917°E	9	0.49	0.29
Puerto Princesa	9.750°N, 118.733°E	8	0.63	0.54
Sandakan	5.817°N, 118.080°E	13	0.63	0.53
Jolo	6.067°N, 121.000°E	4	0.59	0.44
Tawau	4.233°N, 117.883°E	14	0.53	0.52
Listed in the table are the station name and location, record length (years), the overall correlation (Cor1) and correlation with annual cycle removed (Cor2).				

**Table 3:** Climatological monthly root mean square differences (RMSDs) between the drifter observed and the REDOS velocities

**Month**	**Observation Number**	**RMSD of U (cm s** ^**-1**^)	**RMSD of V (cm s** ^**-1**^)	**RMSD of velocity magnitude (cm s** ^**-1**^)
1	8652	16.71	15.76	16.46
2	3622	18.05	14.64	16.71
3	2931	19.06	20.29	21.09
4	2440	18.53	16.90	18.00
5	3627	21.36	19.98	21.70
6	4099	21.13	18.18	19.66
7	4359	21.94	19.45	22.17
8	6340	20.97	27.56	23.24
9	10029	20.28	21.63	23.22
10	13931	16.89	16.76	17.33
11	12996	19.62	18.46	20.67
12	12272	19.52	19.18	20.88
mean	7108	19.51	19.07	20.09
